# Therapeutic potential of herbal topical agents in evidence-based wound care: a systematic research review

**DOI:** 10.1007/s13205-026-05127-w

**Published:** 2026-11-09

**Authors:** Rasna Kumari, Tarun Kumar, Shriram Pandey, Neha Jain, Manisha Pandey, Najwa Mohamad, Mohd Cairul Iqbal Mohd Amin, Pradeep Kumar

**Affiliations:** 1 Department of Pharmaceutical Sciences, Central University of Haryana, Mahendergarh, Haryana 123031 India; 2 Department of Library and Information Science, Central University of Haryana, Mahendergarh, 123031 India; 3 Department of Pharmaceutics, Amity Institute of Pharmacy, Amity University, Noida, India; 4 Department of Pharmaceutical Technology and industry, Faculty of Pharmacy, University of Cyberjaya, Persiaran Bestari, 63000 Cyberjaya, Selangor Darul Ehsan Malaysia; 5 Centre for Drug Delivery Technology & Vaccine, Faculty of Pharmacy, University Kebangsaan Malaysia, Jalan Raja Muda Abdul Aziz, Kuala Lumpur, Malaysia; 6 Wits Advanced Drug Delivery Platform Research Unit, Department of Pharmacy and Pharmacology, School of Therapeutic Sciences, Faculty of Health Sciences, University of the Witwatersrand, 7 York Road, Parktown, Johannesburg, 2193 South Africa

**Keywords:** Herbal dressings, Nanoparticles, Chronic wounds, Infections, Systematic review

## Abstract

Herbal interventions are increasingly investigated for wound infection due to their potential for antimicrobial, antioxidant, anti-inflammatory, tissue-repair and angiogenic activities. Despite this, their clinical relevance is uncertain owing to substantial heterogeneity in botanical materials, phytochemical composition, formulations, experimental models and level of evidence. This systematic review was conducted in accordance with PRISMA 2020. Literature published between 2000 and 2025 was systematically searched in PubMed for original peer-reviewed studies evaluating herbal extracts or purified phytoconstituents, alone or incorporated into topical formulations, for wound infection. In vitro, animal, observational, and clinical studies were included and critically interpreted as per the evidence. Other than this, data on botanical authentication, extraction, standardization, bioactive constituents, wound type, formulation characteristics, and antimicrobial and antifilm activity, efficacy and safety were extracted. The evidence indicated that selected herbal interventions can modulate multiple processes involved in wound repair, including inflammation, oxidative stress, angiogenesis, fibroblast activity, collagen deposition, re-epithelialization, and microbial growth. Nevertheless, substantial heterogeneity in plant sources, extraction methods, concentrations, formulations, wound models, and outcome measures limited direct comparison. Evidence for activity against clinically relevant biofilms and chronic wounds was comparatively limited, while clinical evidence remained less extensive than preclinical evidence. Advanced formulations may improve delivery and wound-site retention, although their independent contribution to therapeutic efficacy requires further clarification. Herbal extracts and phytoconstituents represent potential candidates for topical wound management; however, current evidence does not support their routine replacement of established therapies. Standardized preparations, reproducible formulations, rigorous safety evaluation, clinically relevant chronic-wound models, and adequately powered clinical trials are required for translation into evidence-based wound care.

## Introduction

The skin is highly prone to infections by various agents, which may compromise its structural integrity and result in wounds. The skin is the outermost and largest organ of the human body, alongside muscles, bones, and other internal organs, and ultimately protects the body from chemical, biological, physical, and mechanical factors (Lopes et al. 2024). Generally, wounds are regarded as breaks, or defects, in the skin caused by physicochemical changes and/or thermal damage. Wounds are classified into two types, acute and chronic, based on the healing process. Wounds that take approximately 8–12 weeks to heal completely are known as acute wounds, whereas wounds present in diseases such as cancer, diabetes, and pressure necrosis take longer to heal and are known as chronic wounds. Healing of these types of wounds takes more than 12 weeks to reach a routine healthy state (Suarato et al. 2018). The other type of wound involves skin layers and areas. Wounds on the skin surface are known as superficial wounds, and wounds that impact the epidermis layer are regarded as partial-thickness wounds. In other types of wounds, the subcutaneous layer is injured or interrupted, including hair follicles, deeper dermal layers, sweat glands, and blood vessels (Mir et al. 2018).

Extreme pain, along with the chances of death, are present in case of burn wounds. In addition, extreme pain results in increased restrictions on restoring functionality and cosmetic repair of the patient’s body (Pereira et al. 2014). Based on severity, burns can be divided into two classes acute or chronic. Acute burns result in a sudden influx of growth factors and inflammatory cytokines. Sepsis and septic shock are serious complications of acute burns (Serov et al. 2023).

For several centuries, plant-derived phytoconstituents have been used to treat various types of wounds (Monton et al. 2022), (Carla Daunton et al. 2012). Plant extracts and essential oils have recently attracted attention from the scientific community (Freitas and Cattelan 2018). Essential oils are regarded as secondary metabolites produced from various plant origins, including leaves, seeds, bark, roots, and twigs of different plants and herbs (Negut et al. 2018). They possess antioxidant (Xin et al. 2024)., anti-inflammatory, and antimicrobial properties, and are used in the process of wound healing. Plant extracts are synthesized from natural plants and have several pharmacological properties. In addition, they offer additional advantages, such as cost-effectiveness, efficacy at lower concentrations, lower toxicity, and ease of application (Saepang et al. 2025). These substances modulate ROS generation through intracellular interactions, thereby increasing immune cell responses, reducing inflammation, and accelerating tissue regeneration (Criollo-Mendoza et al. 2023). In this review, we aimed to critically assess and develop documented proof of the scientific findings, effectiveness, and safety of herbal topical dressings (Gupta and Chakraborty 2022), (Fan et al. 2023) for managing infected wounds. By systematically studying a variety of biological and scientific literature, this review explores potential herbal therapies with their therapeutic mechanistic insights and examines their applicability and implementation in advanced wound care strategies.

## Methodology

This study was based on the PubMed database. PubMed is an essential data source for systematic reviews and bibliometric analyses. PRISMA flow was used to gather data on topical and transdermal formulations containing herbal drugs for wound healing and infection. Keywords related to transdermal were used to conduct a search using the following search string:

(“Transdermal Patch” OR “Transdermal Drug Delivery” OR “Skin Absorption Enhancers” OR “Nanoparticles” OR “Drug Design”) AND (“Phytotherapy” OR “Plant Extracts” OR “Anti-Infective Agents” OR “Dosage Forms” OR “Medicine, Traditional” OR “Phytochemicals”) AND (“Wound Infection” OR “Pathogenicity” OR “Bacterial Infections” OR “Biofilms” OR “Wound Healing” OR “Drug Resistance, Microbial” OR “Wounds and Injuries” OR “Infection, Localized”).

The literature was searched from the PubMed database only. The search dates were 4th March 2025 to 25th March 2025. Every record was carefully examined for consistency and complete metadata components, which are crucial for bibliometric analysis. OpenRefine (Ahmi 2023) was utilized to ensure that every record has complete metadata elements by cleaning and harmonizing the datasets. The screened records were limited to English only.

### Systematic literature review

**Fig. 1:**
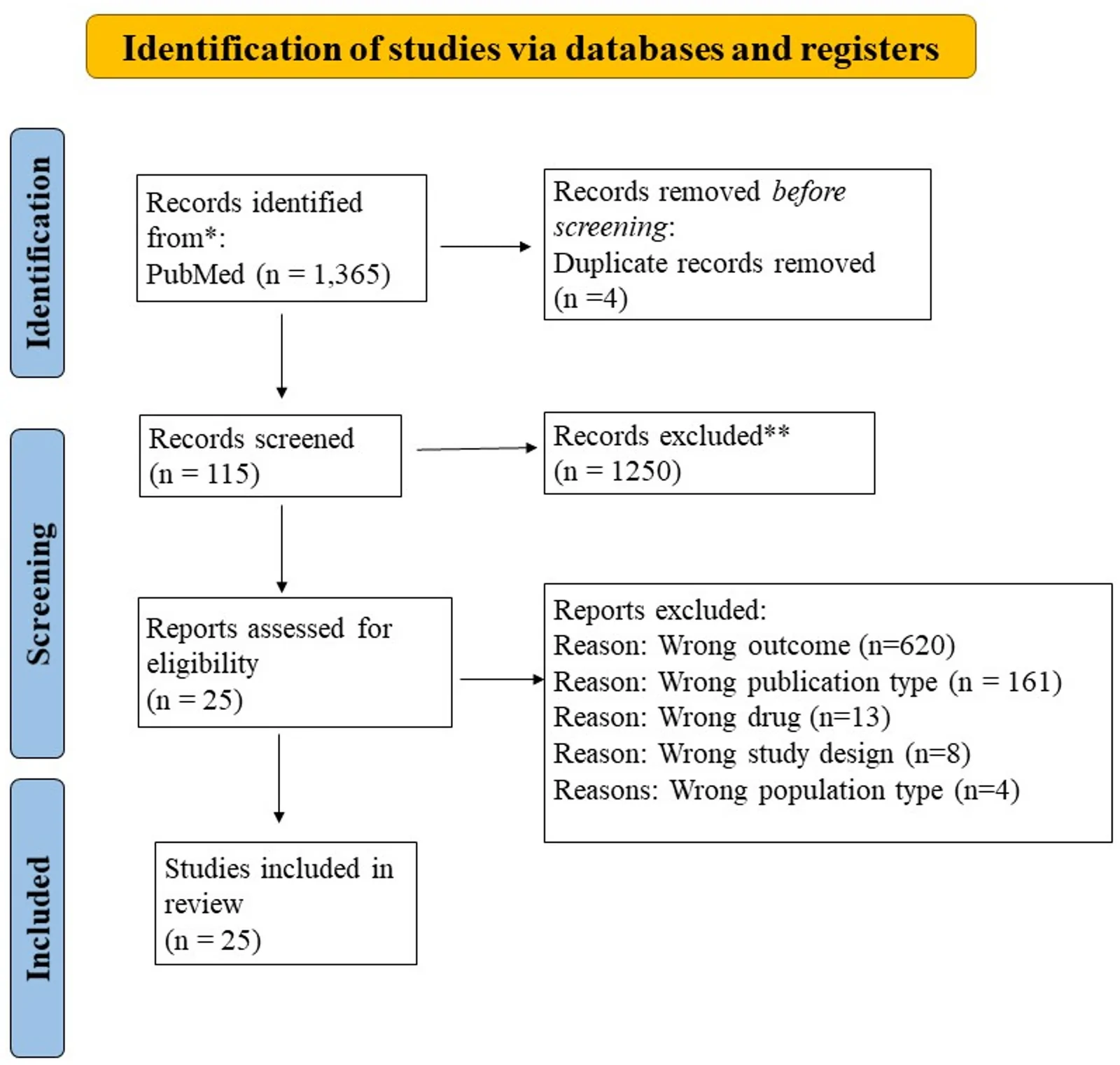
PRISMA flow diagram demonstrates the systematic review’s inclusion and exclusion stages (Page et al. 2021)

The 23 empirical papers were chosen for a systematic literature review (SLR) based on the following criteria:

The inclusion and exclusion criteria for the systematic literature review (SLR) were predefined to ensure consistency and relevance in study selection. The inclusion and exclusion criteria for the systematic literature review (SLR) were predefined to ensure consistency and relevance in study selection. The inclusion criteria comprised: (i) research articles and clinical trials investigating herbal extracts or phytoconstituents for wound healing or wound infection; (ii) studies evaluating topical formulations containing herbal extracts or phytoconstituents for the management of wound infections; (iii) peer-reviewed journal articles; (iv) articles published in the English language to ensure consistency and comprehensibility; and (v) studies published between 2000 and 2025. The exclusion criteria comprised: (i) review articles, systematic reviews, meta-analyses, book chapters, conference proceedings, and other non-original publications; (ii) studies investigating synthetic drugs without a herbal extract or phytoconstituent component; (iii) articles published in languages other than English; (iv) studies evaluating oral or systemic treatment for wound infections without a topical herbal intervention; and (v) studies reporting outcomes unrelated to wound healing or wound infection, including infections at sites other than wounds. The key metrics are shown in Table 1 (Fig. 1).

**Table 1:** Key Metrics

Metric	Value	Insights
Total Publications	113	A solid body of work offers sufficient breadth for pattern analysis.
Mean Citations Per Year	3.61	On average, papers are cited each year moderately, indicating ongoing relevance.
Median Citations Per Year	2.0	Many papers have a modest annual impact, skewed by a few highly cited works.
Mean Total Citations	19.67	This suggests that the overall citation performance is decent.
Median Total Citations	6.0	Most papers are lightly cited, and a few outliers drive up the mean.
Mean RCR	1.76	The average paper performs better than the average NIH-funded publication.
Median RCR	1.14	A typical paper is at or slightly above the benchmark, which is a healthy influence.
Most Frequent Journal	*Spectrochim Acta A Mol Biomol Spectrosc*	Indicates a focus on spectroscopy and biomolecular science.
Proportion Clinical (Yes)	0.00%	Almost no clinical citationindicates a basic or preclinical research focus.

### Keywords co-occurrence cluster analysis

**Fig. 2:**
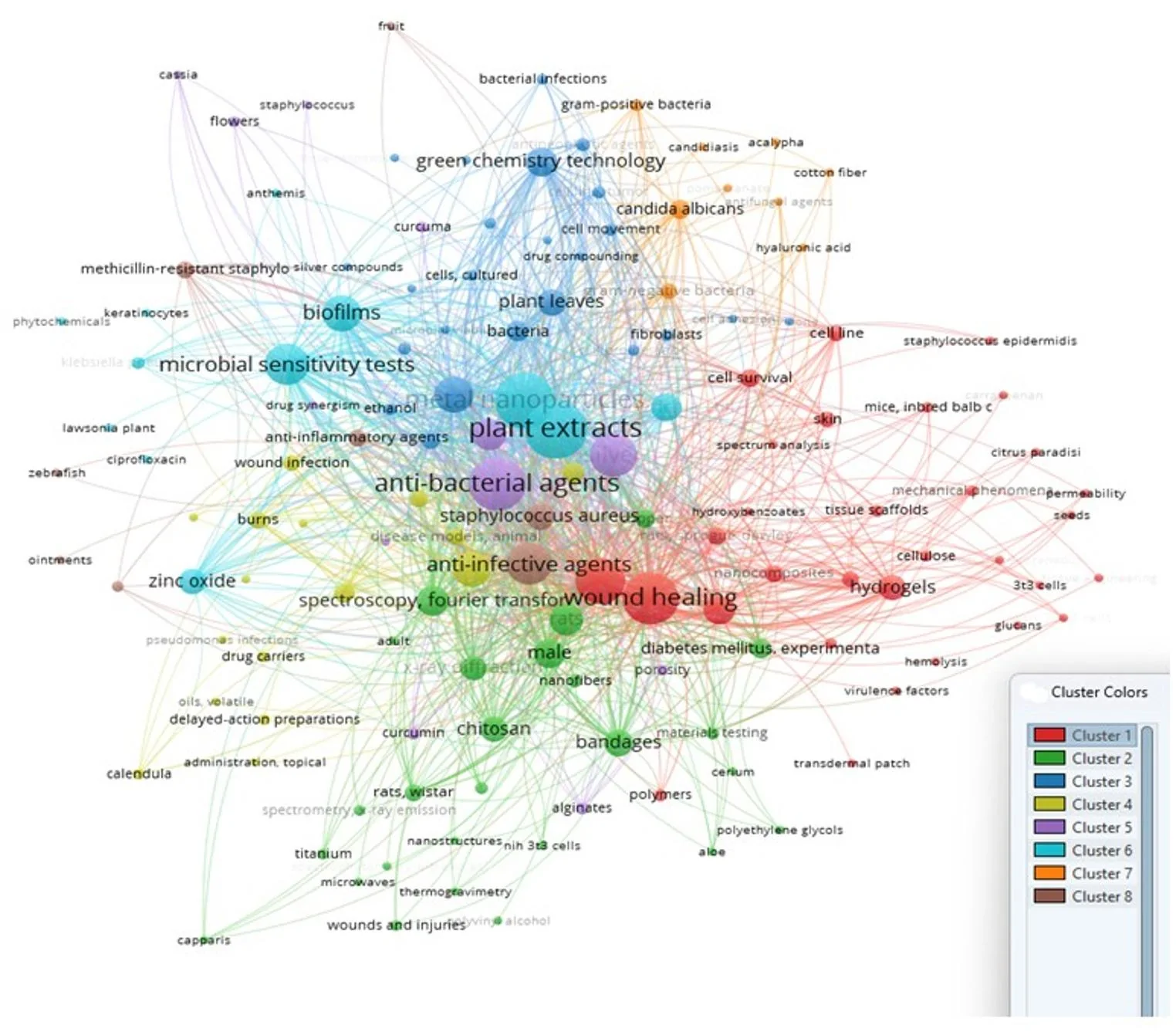
Keyword Co-occurrence Cluster Network Visualization using VOSviewer

The cluster analysis in Fig. 2; Table 2 shows various themes within the theme, highlighting significant focus areas, emerging trends, and foundational concepts. Eight distinct clusters were created from 138 MeSH keywords using a threshold of 2, connected by 2771 edges signifying strong thematic interrelations.

**Table 2:** Cluster Focus and Themes

Cluster #	Keywords	Number of Keywords	Cluster Theme
1	3t3 cells, administration, cutaneous, animals, biocompatible materials, carrageenan, cell line, cell survival, cellulose, citrus paradisi, collagen, glucans, hemolysis, hydrogels, hydroxybenzoates, mechanical phenomena, mice, inbred balb c, nanocomposites, permeability, polymers, rats, raw 264.7 cells, seeds, skin, spectrum analysis, sprague-dawley, staphylococcus epidermidis, tissue engineering, tissue scaffolds, transdermal patch, virulence factors, wound healing	31	Advanced Cellular and Clinical Therapies
2	adult, aloe, bandages, capparis, cerium, chitosan, copper, diabetes mellitus, experimental, male, materials testing, microscopy, electron, scanning, microwaves, nanofibers, nanostructures, nih 3t3 cells, polyethylene glycols, polyvinyl alcohol, rats, rats, wistar, spectrometry, x-ray emission, spectrophotometry, ultraviolet, spectroscopy, fourier transform infrared, thermogravimetry, titanium, wounds and injuries, x-ray diffraction	26	Natural Therapeutics and Traditional Remedies
3	antineoplastic agents, bacteria, bacterial infections, cell adhesion, cell line, tumor, cell movement, cells, cultured, disk diffusion antimicrobial tests, drug, drug compounding, ethanol, female, fibroblasts, green chemistry technology, humans, microbial viability, microscopy, electron, transmission, phytotherapy, plant leaves, dose-response relationship, plants, medicinal, cell proliferation, povidone, silver compounds, time factors	24	Infection Management and Microbial Control
4	administration, delayed-action preparations, topical, burns, calendula, disease models, drug carriers, drug resistance, multiple, bacterial, gold, hydrogen-ion concentration, animal, nanoparticles, nanotechnology, oils, volatile, particle size, P*seudomonas aeruginosa*, *Pseudomonas* infections, wound infection	16	Innovative Topical Formulations
5	alginates, anti-bacterial agents, antioxidants, cassia, curcuma, curcumin, drug synergism, flowers, porosity, sericins, silver, *Staphylococcus*	12	Emerging Biomaterials and Nanotechnology
6	anthemis, biofilms, ciprofloxacin, *Escherichia coli*, keratinocytes, *Klebsiella Pneumoniae*, lawsonia plant, metal nanoparticles, microbial sensitivity tests, phytochemicals, plant extracts, zinc oxide	12	Biofilm and Antibiotic Resistance
7	acalypha, antifungal agents, *Candida albicans*, candidiasis, gram-negative bacteria, cotton fiber gram-positive bacteria, hyaluronic acid, pomegranate	9	Antifungal Treatments
8	anti-infective agents, anti-inflammatory agents, methicillin-resistant, fruit, *Staphylococcus aureus*, ointments, *Staphylococcal* infections, zebrafish, *Staphylococcus aureus*,	8	Chemical Analysis and Drug Synergy

The thematic cluster analysis shows the eight different clusters; each cluster shows a different research topic interconnected with wound healing, such as antimicrobial agents and biomedical applications.

#### Cluster 1: advanced cellular and clinical therapies (TLS: 2925, avg. year: 2020.53)

Cluster 1 is the largest, containing 31 keywords that constitute the heart of the current research, which aims to evaluate advanced clinical methods, including “hydrogels”, “topical administration”, and “animal models.” Its high connectivity and recent publications indicate that researchers are actively investigating treatments using bioengineered tissues and new clinical strategies for effective wound healing.

#### Cluster 2: natural therapeutics and traditional remedies (TLS = 2318, avg. year: 2020.07)

In the second cluster, 26 keywords signify the interest in utilizing nature for healing, containing “aloe”, “chitosan”, “curcumin”, and “phytochemicals.” The strong research interconnections indicate a well-established, actively evolving field in which several natural substances are explored for therapeutic effects, such as antimicrobial activity, strongly reflecting preferences for natural healthcare products.

#### Cluster 3: infection management and microbial control (TLS: 1723, avg. year: 2018.37)

Cluster 3 contains the keywords “bacterial infections,” “Staphylococcus aureus,” and “antineoplastic agents,” which signify foundational, essential research targeting bacterial pathogens.

#### Cluster 4: innovative topical formulations (TLS: 1088, avg. year: 2020.33)

Cluster 4 comprises around 16 keywords, including “burns,” “calendula,” “ointments,” and “nanostructures,” which indicate recent interest in novel topical treatments. Moderate associations highlight the important and focused research addressing clinical limitations in wound care.

#### Cluster 5: emerging biomaterials and nanotechnology (TLS = 1694, avg. year: 2021.96)

Cluster 5 shows 12 keywords, including “nanoparticles,” “antioxidants,” “zinc oxide,” and “polymers,” which are recent and fast-growing areas exhibiting cutting-edge research on innovative antimicrobial and healing materials that reflect technological advances in healthcare.

#### Cluster 6: biofilm and antibiotic resistance (TLS: 2865, Avg. Year: 2021.61)

Cluster 6, having 12 keywords, includes “biofilms,” “ciprofloxacin,” and “Escherichia coli,” which are highly connected and signify intensified research efforts against biofilm formation and antibiotic resistance, underscoring global concerns about resistant infections.

#### Cluster 7: Antifungal Treatments (TLS: 432, avg. year: 2020.28)

Cluster 7 is regarded as a small niche cluster (nine keywords: “*Candida albicans*,” “acalypha”) that shows specialized yet vital research in managing fungal infections, suggesting several growth opportunities as antifungal resistance emerges.

#### Cluster 8: chemical analysis and drug synergy (TLS: 897, avg. year: 2021.09)

Cluster 8 contains eight keywords (“drug synergism,” “spectroscopy,” “ethanol”), which signify an emerging interdisciplinary approach that blends chemistry and pharmacology to enhance therapeutic effectiveness.

### Thematic map

**Fig. 3:**
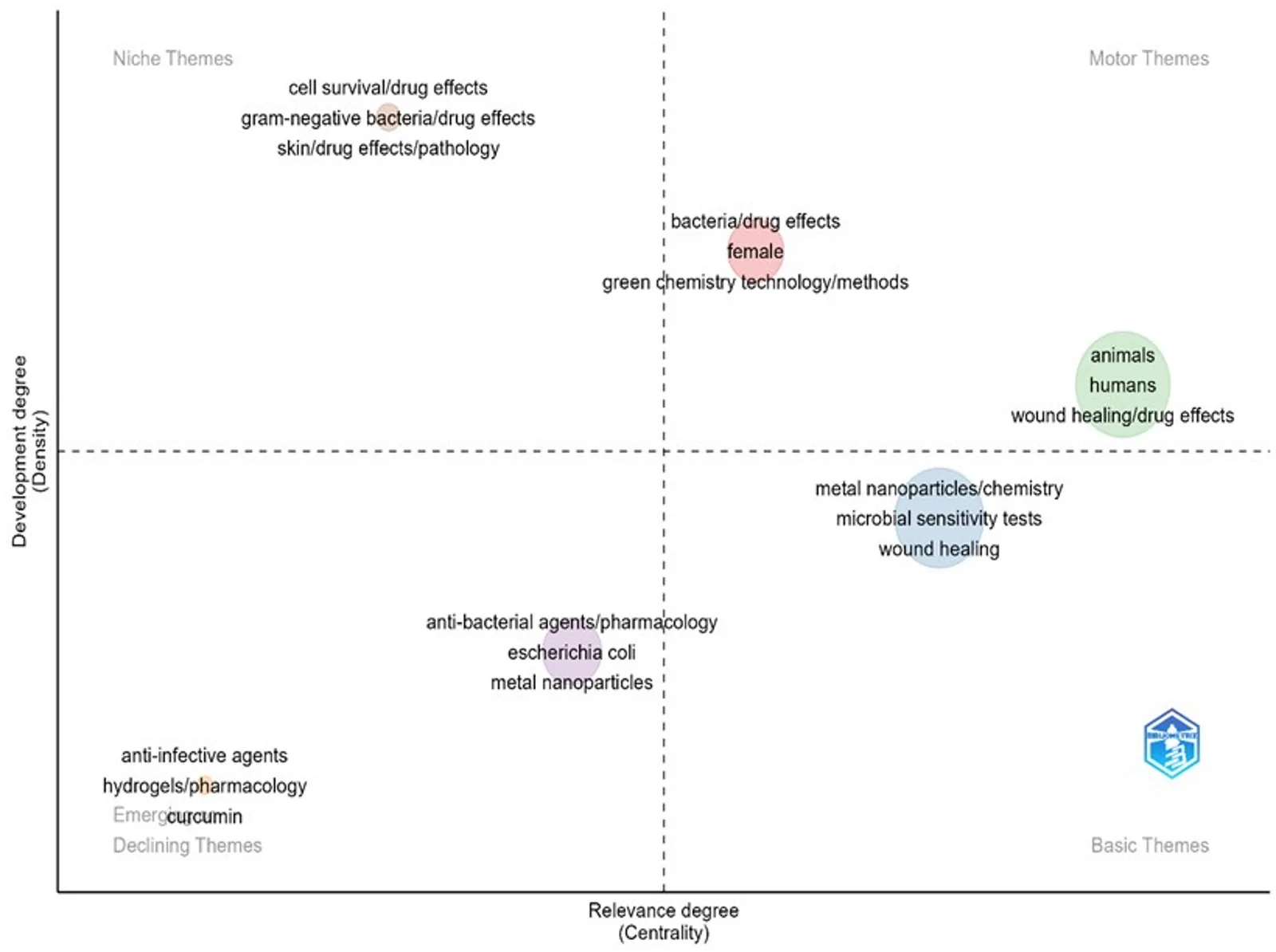
The thematic map depicts a vibrant, emerging research ecosystem with clear clusters of opportunity, influence, and growth prepared by bibliometrix

The major themes in the field are wound healing, animal models, and drug effects in humans. These topics are not well developed, but they are also central to the overall research structure, making them more important in this field. For example, the animal theme scored high centrality (25.80) and density (90.51), with the highest frequency (473), which confirms its dominant role in guiding scientific communication.

Close to this core, *metal nanoparticles/chemistry* and *microbial sensitivity tests* have emerged as basic yet underdeveloped themes. Despite their strong relevance (centrality = 16.31), their moderate density (68.00) recommends the requirement of more cohesive exploration. These are significant areas offering opportunities for future integration and interdisciplinary collaboration.

On the other hand, the very specialized side, with themes such as cell survival/drug effects and gram-negative bacteria, is highly developed but low in centrality, suggesting deep specialization without broad connectivity. These clusters, such as *antibacterial agents/pharmacology* (centrality = 4.99, density = 62.73), are ideal for researchers seeking to carve out impactful expertise in focused subdomains.

**Fig. 4:**
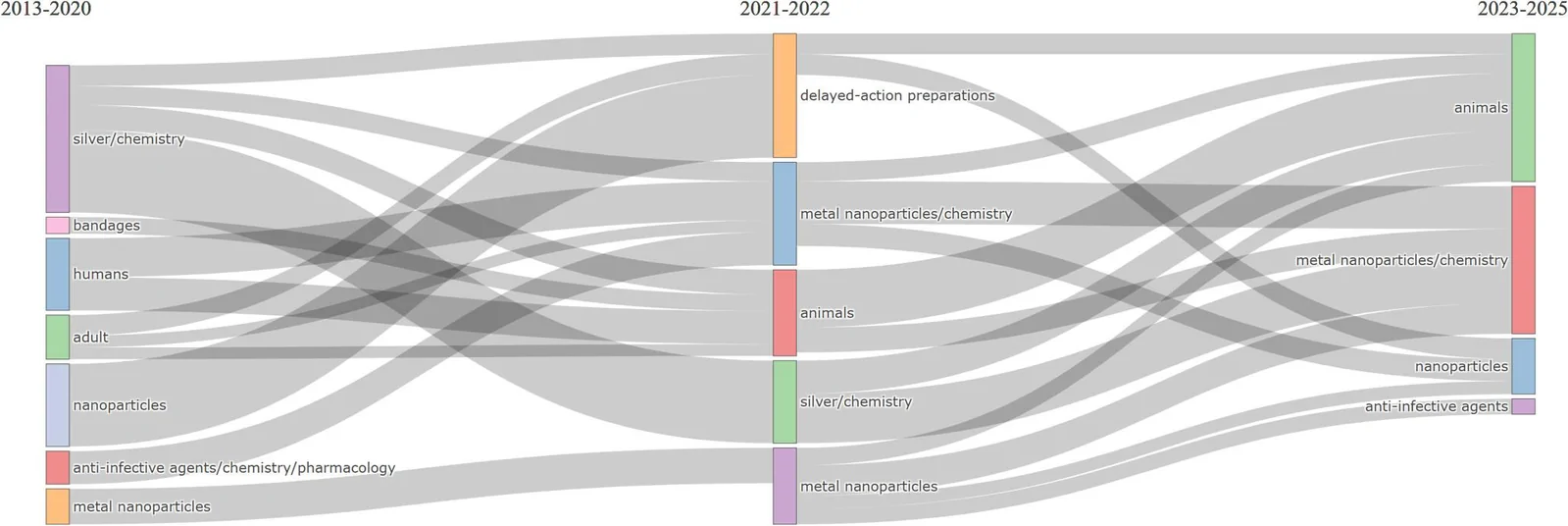
The evolution map shows several research themes that show how the scientific focus has shifted and matured in this domain over time, prepared from Bibliometrix

The themes of *curcumin*, *hydrogels/pharmacology*, and *anti-infective agents* fell into the emerging or declining quadrants. These topics, with the lowest centrality (1.23) and frequency (14), may represent early-stage research or declining interest (Fig. 3 and 4).

Over the past years, research in this field has shifted from foundational biological studies to focused, clinically relevant work. Beginning in 2013–2019, core themes such as “humans,” “animals,” “adult,” “cell survival/drug effects,” and “cultured cells” highlighted the field’s experimental roots. These keywords reflect early efforts to understand biological responses, often using laboratory models to explore wound healing and drug effects.

The 2020–2022 period saw a focus on nanotechnology and antimicrobials. Key emerging themes included “microbial sensitivity tests,” “metal nanoparticles/chemistry,” and “biofilms/drug effects.” The topic *“adult → microbial sensitivity tests”* scored a weighted inclusion index of 0.67, showing a strong thematic link. Likewise, *“animals → metal nanoparticles/chemistry”* and animals *→ gold/chemistry* reflect a transition to nanomaterials in infection control and healing. The 2023–2024 period marks a deeper phase of specialization. Keywords such as “*Escherichia coli*,” “anti-infective agents,” “nanoparticles,” and “cell survival/drug effects” became prominent, indicating a greater focus on pathogens and therapeutic strategies. The keywords “metal nanoparticles/chemistry” remained a persistent theme across all phases.

From 2025, the evolution shifted to a unified clinical theme: “wound healing/drug effects.” This endpoint reflects over a decade of development from biological inquiry and antimicrobial testing to clinically oriented nanomedicine.

## Results and discussion

The selected articles utilized herbal phytoconstituents with dressings, hydrogels, silver nanoparticles, or other nanoparticles embedded in dressings and hydrogels for the treatment and prevention of different types of wound infections.

### Wound type

According to the literature, wound dressings should be evaluated according to the type and pathological characteristics of the wound, and evidence obtained from acute wound models should be distinguished from that obtained from chronic or complicated wound models. In acute wound models, such as experimentally induced excisional or full-thickness wounds in otherwise healthy animals, wound closure primarily reflects the normal healing response and therefore may not accurately predict therapeutic efficacy under chronic pathological conditions. Dressing selection should consequently consider wound characteristics such as exudate level, moisture balance, and infection. For example, sponge dressings may be suitable for low-to-medium exudative wounds, with a reported water vapor transmission rate (WVTR) of 1516.81 ± 155.25 g/m²/day (Kaur Sandhu et al. 2023). Similarly, WVTR requirements can vary considerably according to wound type, ranging from 279 to 5138 g/m²/day in different wound conditions, including first-degree burns and granulating wounds (Kaur Sandhu et al. 2023). However, the efficacy demonstrated in such acute wound models should not be directly extrapolated to chronic wounds, where persistent inflammation, impaired angiogenesis, infection, oxidative stress, and metabolic dysfunction can substantially delay healing. Therefore, studies using pathological wound models provide more relevant evidence for evaluating the therapeutic potential of advanced wound dressings. For example, Bao et al. (Bao et al. 2024)evaluated a porcine acellular dermal matrix hydrogel containing curcumin and magnesium-doped bioactive glass nanoparticles (PADM@MgC) in an *S. aureus* infected excisional wound model and demonstrated improved wound healing together with antibacterial and inflammation-regulating effects. This infected wound model provides evidence of dressing performance under conditions that more closely resemble the challenges associated with infected wounds than an uncomplicated acute wound model. Likewise, full-thickness diabetic wound models have been used to evaluate lignin-based hydrogels under metabolically compromised conditions, providing evidence that is more relevant to chronic diabetic wound healing (Fadilah et al. 2023). Thus, the therapeutic efficacy of wound dressings should be interpreted in the context of the wound model used, with findings from acute wounds considered separately from those obtained in diabetic, infected, ischemic, or other chronic wound models.

### Sources of variability and inconsistency among experimental wound-healing studies

The inconsistent findings reported among wound-healing studies may partly arise from substantial differences in experimental design, including the wound model, treatment regimen, wound assessment method, and duration of follow-up. Variations in wound etiology, wounding procedure, treatment conditions, wound assessment, and data analysis can influence the interpretation and reproducibility of preclinical wound-healing outcomes (Ojeh et al. 2025). The experimental wound model is an important source of variability because incisional, excisional, burn, infected, diabetic, and other impaired-healing models reproduce different aspects of the wound-healing process. Differences in animal species and strains, wound location, wound depth, wound size, and whether healing occurs by contraction or re-epithelialization can consequently produce different responses to the same therapeutic intervention (Sorg et al. 2017), (Saeed and Martins-Green 2023). Treatment concentration is another important factor that may contribute to inconsistent outcomes. Differences in the concentration or dose of an active compound can alter its biological activity, toxicity, tissue penetration, and interaction with the wound microenvironment; therefore, apparently similar formulations may produce different healing responses when administered at different concentrations (Ojeh et al. 2025). Similarly, the frequency and duration of treatment administration may influence therapeutic efficacy. A treatment administered once daily, intermittently, or continuously may generate different exposure levels at the wound site, while differences in treatment initiation and treatment duration can affect the stage of healing at which the therapeutic agent exerts its effects. Such differences in treatment conditions should therefore be considered when comparing results across studies (Ojeh et al. 2025). Differences in wound-size measurement methods can also contribute substantially to apparent discrepancies between studies. Wound area may be assessed using a ruler, manual planimetry, digital planimetry, photographic image analysis, or three-dimensional techniques, and these approaches differ in accuracy, reliability, and susceptibility to observer-related variation (Jørgensen et al. 2016). Importantly, even within the same experimental wound model, the choice of assessment method can influence the observed healing response. Studies comparing planimetric and histological assessments have demonstrated that different evaluation approaches may produce different patterns of healing, emphasizing the importance of using standardized and complementary outcome measures when comparing therapeutic effects (Ansell et al. 2014), (Ojeh et al. 2025). The timing of outcome assessment and follow-up period is another major source of heterogeneity. Wound healing is a dynamic process involving inflammation, proliferation, re-epithelialization, granulation tissue formation, angiogenesis, and remodeling; therefore, measurements obtained early point may capture a different biological response than measurements obtained during later stages of healing (Saeed and Martins-Green 2023). For example, an intervention that primarily accelerates early inflammatory resolution or re-epithelialization may show a significant effect during the initial days after wounding, whereas its influence on collagen remodeling, tissue maturation, or scar formation may only become evident during longer follow-up. Consequently, studies using different observation periods may reach different conclusions regarding the overall efficacy of the same treatment (Ojeh et al. 2025). Taken together, differences in wound model, treatment concentration, administration frequency, wound-size measurement technique, assessment parameters, and follow-up duration represent important sources of heterogeneity that should be considered when interpreting apparently inconsistent findings across wound-healing studies. Standardized reporting of these experimental variables is therefore essential to improve reproducibility, facilitate comparison between studies, and strengthen the translational relevance of preclinical wound-healing research (Ojeh et al. 2025).

### Innovative topical formulations and emerging biomaterials with nanotechnology

Different types of topical wound dressings are discussed in the reviewed literature. Passive dressings are best for dry wounds and promote clot formation by absorbing blood and exudate, which can ultimately cause skin tearing and wound reopening when removed. While shielding the wound from additional harm and infection, contemporary wound dressings are made to promote natural healing and re-epithelialization. These dressings have several benefits, including maintaining a moist environment, absorbing excess extracellular fluid, creating an infection barrier, keeping the wound at the proper temperature, easing discomfort, promoting epidermal migration, and reducing medical expenses. Other types of wound dressings include curcumin-loaded solid lipid nanoparticles (CSLNs) and probiotics (*Lactobacillus pantarum*) in combination with wound dressings. The dressing has anti-infective, analgesic, anti-inflammatory, and antioxidant properties. CSLNs aim to promote the bio-profile of curcumin while achieving controlled release (Kaur Sandhu et al. 2023).

Hydrogels and nanofiber films are also types of wound dressings. The major characteristics of hydrogels are their permeability, moisturizing properties, and biocompatibility. It initially releases curcumin to inhibit infection and inflammation by damaging bacterial cell membranes, followed by Mg^2+^ to form an anti-inflammatory microenvironment, resulting in angiogenesis and tissue repair (Bao et al. 2024). Lignin-based hydrogels loaded with PAS/SL@AgNPs are also available and accelerate wound healing, offering antioxidant, antimicrobial, and tissue-fluid-removal capabilities. Their three-dimensional network structure, antioxidant activity, antibacterial qualities, tissue fluid removal capacity, free radical scavenging, and antimicrobial activity are all credited with their efficacy (Jin et al. 2025). Sulfur nanoparticles (SNP) and *Garcinia esculenta* (GSE) extract were incorporated into carrageenan-based hydrogel films for wound-healing testing, which showed a remarkable reduction in wound size (Jaiswal et al. 2019). Green manufactured zinc oxide nanoparticles based on keratin (K)-sodium alginate, and the herbal remedies *C. roseus* and *M. recutita* are used in another wound dressing. Sustained drug release, thermal stability, enhanced biocompatibility, superior antibacterial activity, water vapor transmission, and oxygen permeability are among these dressings’ advantages. Medicated cotton-gauze was also used as a control in the experiment to evaluate keratin-sodium alginate-based dressings (Sellappan and Manoharan 2024). Chitosan hydrogels loaded with AgNPs and Calendula extract are used in skin injuries for cicatrization (scar formation). These can absorb water and keep ulcers hydrated, and they have antibacterial and anti-inflammatory activities. Another study investigated *Garcinia esculenta* extract-loaded sulfur nanoparticles embedded in carrageenan-based hydrogel films for wound healing. The superior healing efficiency of the 3% SNP formulation was attributed to the high concentration of SNPs, with sulfur’s role in forming hydrogen sulfide, which induces keratosis and causes histological changes for skin restoration. Carrageenan hydrogel, with its porous structure and GSE properties, also contributes to wound healing (Jaiswal et al. 2019).

### Natural therapeutics and their mechanism for wound healing

#### Plant extracts

There are so many plants that are used traditionally for the prevention and cure of different health conditions. Similarly, different plant extracts are used for the cure of wounds. *Chromolaena odorata* is a medicinal plant used in the formation of the transdermal patch to increase the wound-healing properties. The leaf extract was traditionally used to stop bleeding from wounds and to heal external wounds. Additionally, it induces the expression of genes related to wound healing and homeostasis, thereby promoting fibroblast cell migration and proliferation (Latif et al. 2024). *Gliricidia sepium* leaf extract contains the phytoconstituents apigenin-7-O-glucoside and kaempferol, along with protocatechuic acid, which have anti-inflammatory activity. It is used in the development of a biogenic nanohydrogel incorporating zinc oxide nanoparticles for the healing of diabetic wounds. The hydrogel promoted tissue regeneration, reduced apoptosis, and modulated inflammation in diabetic wounds (Wafaey et al. 2025). Leaf extracts of *Catharanthus roseus* and *Matricaria recutita*, Chamomile flower parts, were used to synthesise green zinc oxide nanoparticles that are loaded into keratin-sodium alginate wound dressings. *C. roseus* has anti-inflammatory and antibacterial properties, whereas Chamomile contains phytoconstituents like apigenin and bisabolol that promote wound healing (Sellappan and Manoharan 2024). Aloe vera extract is a well-known wound-healing agent that is incorporated into chitosan-based nanocomposites containing biogenic silver nanoparticles (Sathiyaseelan et al. 2017). *Calendula officinalis* is also a well-known anti-inflammatory agent that stimulates phagocytosis and promotes granulation by affecting glycoprotein, nucleoprotein, and collagen metabolism (Rodríguez-Acosta et al. 2022). Astragalus *membranaceus* (Huang Qi) is a traditional Chinese medicine that is effective in diabetic foot ulcers. The major components involved in immunomodulatory functions and in enhancing cellular metabolism are astragalus polysaccharides (APS) and astragaloside IV (AS) (Yue et al. 2022). *Cydonia oblonga* extract is a medicinal plant with antibacterial, antioxidant, and wound-healing potential. The plants are effective against both Gram-positive and Gram-negative bacteria (Ashtariyan et al. 2024). Corn silk extract (CSE) is used in the thermosensitive hydrogels, along with hyaluronic acid and nanosilver, for wound healing (Ashtariyan et al. 2024). There are several parameters that should be considered before using any kind of medicinal plant. The list of few parameters is listed in the Table 3.

**Table 3:** Geographical Origin, Harvesting Conditions, Extraction Methods, and Phytochemical Profile of Selected Medicinal Plants

Plant species	Origin	Harvesting	Extraction methods	Active constituents	References
*Chromolaena odorata*	Native to tropical & subtropical America; now widely distributed in Africa and tropical Asia, including India.	Mainly young/fresh leaves	Aqueous, ethanol, hydroethanolic and fractionated extracts	Flavonoids, phenolic compounds, terpenoids, alkaloids, tannins; major reported compounds include quercetin	(Li et al. 2024)
*Gliricidia sepium*	Native mainly to Mexico–Central America/Colombia; now cultivated widely in tropical regions.	Primarily leaves	Aqueous/ethanolic and other solvent extracts	Flavonoids, saponins, tannins, terpenoids, glycosides, phenolic compounds	(Aulanni’am et al. 2021)
Catharanthus roseus	Native to Madagascar, especially eastern and southern Madagascar; now cultivated/naturalized throughout tropical regions.	Leaves, flowers and aerial parts, the material is generally collected during active vegetative/flowering growth	Aqueous, ethanol, methanol and hydroalcoholic extracts	Monoterpenoids indole alkaloids including vincristine, vinblastine, vindoline and catheranthine; also flavonoids, phenolics and terpenoids	(Khan et al. 2025)
*Matricaria recutita*	Native from Europe to Afghanistan and northern India (Punjab); now cultivated widely in Europe, India, Egypt, USA and other countries.	Flower heads/capitula usually harvested when flowers are fully open	Mainly aqueous infusion/decoction, ethanol/hydroalcoholic extracts and essential-oil extraction	Apigenin, apigenin-7-glucoside, luteolin, quercetin, α-bisabolol, bisabolol oxides, chamazulene	(Cheimpeloglou et al. 2026)
Aloe vera	Origin associated with the Arabian Peninsula; now cultivated worldwide.	Mature leaves, particularly inner leaf fel, harvested mature leaves are processed rapidly to obtain gel	Hydroalcoholic extracts	Acemannan/acetylated mannas, glucomannan, polysaccharides, aloin/aloe-emodin (maily latex), chromones, phenolics, vitamins and amino acids	(Abid et al. 2025)
*Astragalus membranaceus*	Indigenous mainly to northern/eastern China and Mongolia.	Dried roots are the principal medicinal material	Water decoction, aqueous polysaccharide extraction and chromatographic isolation	Astragalus polysaccharides (APS), astragaloside IV, flavonoids, isoflavonoids and saponins	(Liu et al. 2024)
Calendula officinalis	Native mainly to Western Mediterranean/Southern Europe; widely cultivated worldwide.	Flowers/inflorescences are most frequently used. Flower developmental stage and cultivar affect flavonoid content.	Infusion, aqueous extract, ethanol/hydroalcoholic extract, tincture, oily extract	Triterpenoids, flavonoids, carotenoids, phenolic acids, coumarins, volatile oils and calendic acid	(Ejiohuo et al. 2024)
Cydonia oblonga (quince)	Native range Caucasus to Central Asia, including Iran, Iraq, Transcaucasia, Tajikistan and Uzbekistan.	Fruit pulp, peel and seeds are extensively investigated; leaves are also studied. Composition varies according to tissue and cultivation	Aqueous extraction of seed mucilage	Quince seed mucilage, mainly polysaccharides/glucuronoxylan; phenolics and flavonoids occur in other plant parts	(Erikçi et al. 2026)

#### Phytochemicals

The plant extracts contain several bioactive compounds that are mainly responsible for the pharmacological outcome following by several mechanisms. The first and well-known phytoconstituent is Curcumin, a polyphenol compound with several pharmacological properties, including anti-infectious, antioxidant, anti-inflammatory, and analgesic activities. It is reported that it enhances re-epithelialization, raises the number of fibroblasts and macrophages, promotes myofibroblast migration, and enhances neovascularization along with collagen deposition.

#### Mechanism

The effectiveness of herbal topical therapies is closely influenced by the wound microenvironment, particularly in chronic and diabetic wounds. Persistent inflammation is frequently accompanied by excessive production of reactive oxygen species (ROS), which can damage cellular membranes, proteins and extracellular-matrix components and further delay tissue repair as mentioned in the Table 4 (Xiang et al. 2024). Phytoconstituents such as curcumin, quercetin, kaempferol, asiaticoside and madecassoside may counter this environment through antioxidant and anti-inflammatory mechanisms, thereby facilitating the transition from inflammation to the proliferative phase. Inadequate vascularization and persistent hypoxia can additionally restrict oxygen and nutrient availability and impair fibroblast activity, collagen deposition and re-epithelialization (Chanu et al. 2023). Compounds such as asiaticoside and madecassoside have demonstrated pro-angiogenic effects, while curcumin has been associated with modulation of VEGF and other angiogenic pathways. Excessive matrix metalloproteinase (MMP) activity represents another important feature of chronic wounds because accelerated degradation of extracellular-matrix proteins can prevent stable tissue formation. Certain phytochemicals can modulate MMP/TIMP balance and thereby influence extracellular-matrix turnover. Furthermore, bacterial colonization and biofilm formation can maintain chronic inflammation and interfere with re-epithelialization. Consequently, the antimicrobial activity of some phytoconstituents may complement their anti-inflammatory, antioxidant and tissue-regenerative effects. Nevertheless, most mechanistic evidence remains preclinical, and the ability of individual phytochemicals or complex plant extracts to simultaneously correct these pathological microenvironmental factors requires further validation in standardized clinical models (Goswami et al. 2023).

**Table 4:** List of plant extracts/phytoconstituents and their mechanism in wound healing

Plant extract/Phytoconstituents	Wound-healing process	Molecular mechanism	Effect on wound microenvironment	References
*Chromolaena odorata* leaf extract	Hemostasis, Inflammation and Proliferation	Modulates genes associated with wound healing and homeostasis and promotes fibroblast migration and proliferation; traditionally associated with hemostatic activity.	Facilitates early bleeding control and transition toward fibroblast-mediated tissue formation and wound closure	(Pandith et al. 2013)
*Gliricidia sepium* leaf extract	Proliferation, inflammation and tissue regeneration	Contains apigenin-7-O-glucoside, kaempferol and protocatechuic acid; associated with modulation of inflammatory responses and tissue regeneration.	Reduces excessive inflammation and apoptosis while supporting regeneration in diabetic wounds.	(Wafaey et al. 2025 a)
*Catharanthus roseus* leaf extract	Inflammation/infection control	Exhibits anti-inflammatory and antibacterial properties; its incorporation into ZnO nanoparticle-containing dressings provides an antimicrobial component.	May reduce microbial burden and excessive inflammation, thereby facilitating subsequent proliferative repair.	(Malaiappan et al. 2024)
*Matricaria recutita* (chamomile) leaf extract	Inflammation/proliferation	Contains bioactive constituents, including apigenin and bisabolol, with anti-inflammatory and antioxidant properties that may support tissue repair.	Helps attenuate inflammatory responses and oxidative stress, supporting wound closure.	(Lairikyengbam et al. 2024)
Aloe vera extract	Inflammation, proliferation and re-epithelialization	Polysaccharides and other constituents promote cell proliferation/migration and provide anti-inflammatory and antimicrobial effects.	Supports wound closure and cellular regeneration while limiting microbial contamination.	(Iosageanu et al. 2024)
*Calendula officinalis* extract	Inflammation, granulation and ECM formation	Exhibits anti-inflammatory activity and stimulates phagocytosis and granulation, with effects on glycoprotein, nucleoprotein and collagen metabolism.	Promotes clearance of damaged material and formation of granulation tissue and ECM.	(Preethi and Kuttan 2009)
Corn silk extract (CSE)	Infection control/ Proliferation	Incorporated into thermosensitive hydrogels with hyaluronic acid and nanosilver; contributes to biological activity while nanosilver provides strong antibacterial action	Reduces bacterial burden and supports wound closure	(Makvandi et al. 2019)
Protocatechuic acid	Inflammation / oxidative stress	Suppresses TLR4-dependent Akt/mTOR, NF-κB, JNK and p38-MAPK signaling, reducing inflammatory mediators such as TNF-α, IL-1β, IL-6, COX-2 and iNOS.	Reduces excessive inflammatory signaling and oxidative stress that can prolong the inflammatory phase.	(Nam and Lee 2018)
Kaempferol	Inflammation / oxidative stress	Modulates NF-κB/MAPK signaling and decreases inflammatory mediators; antioxidant activity helps regulate excessive ROS.	Limits persistent inflammation and oxidative injury, supporting progression toward proliferation.	(Park et al. 2009)
Apigenin-7-O-glucoside	Inflammation / oxidative stress	Reported to modulate NF-κB/NLRP3/caspase-1 inflammatory signaling and oxidative stress.	May reduce macrophage-mediated inflammation and oxidative damage in the wound microenvironment.	(Zhang et al. 2024)
Curcumin	Inflammation / oxidative stress, ECM, remodeling, Microbial/ biofilm control	Suppresses inflammatory signaling including NF-κB and reduces pro-inflammatory cytokines; scavenges ROS and improves redox balance. Enhances collagen deposition, wound contraction and tissue remodeling; may regulate MMP/TIMP and TGF-β-related pathways. Also, Demonstrates antibacterial, antifungal and reported antibiofilm activity.	Helps resolve excessive inflammation and oxidative stress, particularly relevant to chronic and diabetic wounds. May reduce microbial load and biofilm-associated persistence, thereby facilitating wound repair.	(Panchatcharam et al. 2006)

### Antimicrobial study and biofilm formation

The studies identified in the literature employed several in vitro approaches to evaluate antimicrobial activity, including zone of inhibition assays, determination of minimum inhibitory concentration (MIC) and minimum bactericidal concentration (MBC), plate-counting methods, scanning electron microscopy (SEM), live/dead staining, and assessments of bacterial membrane damage and leakage of intracellular contents. The microorganisms investigated encompassed a broad range of Gram-positive and Gram-negative bacteria, as well as fungi relevant to wound infections. Collectively, these studies demonstrate the promising antimicrobial potential of nanoparticles, particularly silver nanoparticles (AgNPs) and zinc oxide nanoparticles (ZnO NPs), as well as selected phytoconstituents, plant extracts, and probiotics. Representative examples include AgNPs (Fernandes et al. 2023), curcumin (Maghimaa and Alharbi 2020), *Lactobacillus plantarum* (Kaur Sandhu et al. 2023), quercetin (Badhwar et al. 2021), lignin-based hydrogel (Jin et al. 2025), PADM@MgC hydrogel (Bao et al. 2024), keratin-based chitosan biopolymeric mats incorporating ZnO nanoparticles (Sellappan and Manoharan 2024), grapefruit seed extract (GSE) (Koneru et al. 2020), and tannic acid nanoparticle hydrogel (Rafatou et al. 2019). These findings indicate that such agents may contribute to microbial control through mechanisms including inhibition of microbial growth, disruption of cell membranes, leakage of intracellular components, and alteration of bacterial morphology or biofilm-associated structures. However, the majority of the available evidence is derived from in vitro studies and experimental formulations, and antimicrobial activity demonstrated under laboratory conditions cannot be directly equated with clinical efficacy against wound infections. Therefore, these herbal agents and phytoconstituents should currently be considered complementary or adjunctive approaches to conventional antimicrobial therapy rather than replacements, particularly in clinically established, severe, or systemic wound infections. Further standardized preclinical studies and well-designed clinical trials are required to establish their therapeutic efficacy, optimal dosage, safety, and potential role as standalone antimicrobial interventions. On the other hand, biofilm formation is a critical feature of chronic and infected wounds because microorganisms embedded within an extracellular polymeric matrix exhibit enhanced tolerance to antimicrobial agents and host immune responses. Therefore, the therapeutic relevance of herbal agents should be evaluated not only against planktonic bacteria but also against biofilm-associated microorganisms. Among the plants discussed in this review, *Chromolaena odorata* has demonstrated antimicrobial and antibiofilm potential, with recent evidence indicating that its ethanolic extract exhibits activity against clinically relevant bacteria and can interfere with biofilm formation; however, earlier studies predominantly evaluated planktonic bacterial growth, highlighting the need for more standardized biofilm models and mechanistic investigations (Phetburom et al. 2025). *Catharanthus roseus* has also shown direct antibiofilm activity against biofilm-forming MRSA and *Candida albicans*, supporting its relevance beyond conventional planktonic antibacterial assays (Neglo et al. 2022). Similarly, *Matricaria recutita* (chamomile) hydroalcoholic extract was shown to reduce established C. albicans and Enterobacter cloacae biofilms, although the extent of activity was dependent on the microorganism and extract concentration (PEQUENO et al. 2018). Collectively, these findings indicate that herbal agents may act through multiple complementary mechanisms, including inhibition of microbial adhesion and biofilm formation, disruption of established biofilms, antimicrobial activity, attenuation of inflammation and promotion of tissue repair; however, the current evidence is heterogeneous, and future studies should prioritize standardized assays of biofilm biomass, viable biofilm cells, biofilm eradication, extracellular polymeric substance disruption and activity against clinically relevant polymicrobial wound biofilms rather than relying predominantly on planktonic growth inhibition assays.

### In vivo wound healing capacity

According to the sources, the Lacca mice utilized in the study had S. aureus-induced wounds approximately 6 mm in diameter. On the 12th day of the trial, the combination sponge reduced the wound by 99 ± 1.51%; the blank sponge, CSLN sponge alone, and L. plantarum sponge alone were also better than the positive control. Bao et al., (Bao et al. 2024)mention PADM@MgC Hydrogel for infected wound healing. In this study, male ICR mice weighing 18–20 g and 4–5 weeks old are used, with an induced excisional skin infection measuring almost 8 mm in diameter with *S. aureus* (2 × 10⁸ CFU/mL). It is observed that wound closure is enhanced, particularly in the initial phases of healing, in the group treated with PADM@MgC. Qualitative assessment shows a higher contraction rate than other PADM@MgC group members and a decreased bacterial survival rate. Badhwar et al., (Badhwar et al. 2021)discuss the QCT-AgNPs hydrogel for diabetic wound healing. Diabetic induced 25–35 g in 8–10 weeks old, Swiss albino mice) with STZ-induced diabetes (blood glucose ≈ 250 mg/dL). Excision wounds of 9 mm diameter and 3 mm depth were formed. The hydrogel shows significant wound closure in diabetic mice in comparison with the marketed gel and the diabetic control group.

Latif et al. (Latif et al. 2024) used a Chromolaena odorata-layered Nitrile Rubber Polymer Transdermal Patch in rats with excision wounds. The *C. odorata* wounds exhibit larger fibrin-filled spaces and greater neutrophil infiltration than the negative controls. For the determination of the wound healing stage, macroscopic observation and wound contraction percentages were used. As per Sellappan and Manoharan (Sellappan and Manoharan 2024), dual-drug wound dressing biopolymeric mats were used, in which male Albino Wistar rats of 190–240 g with full-thickness excision wounds (1.5 × 1.5 cm²) were used. This shows smoother, scarless closure with higher wound closure compared with the control group on days 5, 10, and 15. Jaiswal et al., (Jaiswal et al. 2019) used κ-Carrageenan-based Hydrogel Films with GSE and Chitosan Capped Sulfur Nanoparticles (SNP) in male Sprague-Dawley rats who were six weeks old and had full-thickness 8 mm wounds. The results showed an excellent wound-healing effect, i.e., 1.3% wound area after 2 weeks, compared with the control group, which was 31% wound area. (Jin et al. 2025) used Lignin-based hydrogels along with silver nanoparticles in mice with a skin wound model on their dorsal necks. The observation found accelerated wound healing when compared with the control. The hydrogel group showed the most effective wound healing, achieving 96.37% efficiency by the 21st day. (Preethi et al. 2020)used silver nanoparticle-loaded hydrogel scaffolds in male BALB/c mice of 20–25 g with excisions of full thickness of 5 mm. Within 14 days, all wounds healed. Jiang et al., (Jiang et al. 2023)mention the sodium alginate/Chitosan Sponges Loaded with Sericin-AgNPs and curcumin in male Sprague-Dawley rats of 200–220 g with the full-thickness circular wounds of 10 mm diameter with *S. aureus* or *P. aeruginosa.* The results show the best wound healing in both microorganisms, with the smallest wound area and the highest healing ratio over 12 days. Yue et al., (Yue et al. 2022) used electrospun Nanofibre membranes for diabetic wound healing in diabetic rats with skin wounds. The optimal wound repair effect was observed, with the largest wound-healing area on day 15 (94.5 ± 6.1%), compared with other groups.

### Physical and functional properties of topical wound-healing formulations

Hydrogels, sponges, films, and other advanced wound-dressing systems are important components of topical wound management because they provide physical protection while creating a favorable microenvironment for tissue repair. Their therapeutic performance depends not only on the biological activity of incorporated herbal extracts or phytoconstituents but also on physicochemical properties such as wound-surface adhesion, moisture retention, exudate management, mechanical stability, controlled release, tissue interaction, and ease of application (Garg et al. 2025). Recent reviews have emphasized that these properties are critical for designing functional wound dressings capable of protecting the wound, maintaining hydration, delivering bioactive compounds, and supporting tissue regeneration (Wang et al. 2024b), (Liu et al. 2025). Hydrogels are particularly advantageous because their high-water content enables them to maintain a moist wound environment, which supports cell migration, proliferation, re-epithelialization, and tissue regeneration. However, effective moisture management requires an appropriate balance between water retention and water-vapor transmission to avoid excessive hydration and maceration of the surrounding tissue (Wang et al. 2024a) (Alberts et al. 2025). In addition, dressings should possess adequate exudate-handling capacity to absorb and retain wound fluid while maintaining a suitable moisture balance. This is particularly important for highly exuding wounds, where insufficient exudate management may result in leakage and periwound skin damage (Geri et al. 2025). Adhesion and retention at the wound surface are also important formulation characteristics. Adequate adhesion can maintain intimate contact between the dressing and wound bed, improve therapeutic retention, and facilitate delivery of bioactive compounds; however, excessive adhesion may damage newly formed tissue and cause pain during dressing removal. Therefore, modern hydrogel systems aim to achieve an appropriate balance between adhesion and non-adhesion, particularly under the wet conditions of a wound (Wang et al. 2024a) (Cui and Li 2025). Hydrogels can also function as extracellular-matrix-like scaffolds that support cellular interactions, tissue regeneration, and granulation-tissue formation. Their composition, porosity, swelling behavior, and cross-linking density can be tailored to regulate mechanical properties and the transport of therapeutic molecules within the wound environment (Zhang et al. 2025b), (Gao et al. 2025). Another important advantage is their ability to serve as controlled-release platforms for herbal extracts, phytoconstituents, antimicrobials, nanoparticles, growth factors, and other therapeutic agents. The three-dimensional polymeric network of hydrogels can regulate the diffusion and release of incorporated agents, thereby prolonging their local availability and potentially reducing rapid loss from the wound surface. The release behavior can be modified through polymer composition, cross-linking density, degradation rate, and responsiveness to wound-associated stimuli such as pH or enzymes (Wang et al. 2024a), (Cui and Li 2025). Formulation stability and mechanical integrity are equally important for maintaining dressing performance during storage and application. Changes in swelling, degradation, structural integrity, or premature release of the active component can affect therapeutic efficacy. Recent hydrogel studies have therefore focused on self-healing, mechanical stability, biodegradability, and stimulus-responsive properties to improve the durability and functionality of wound dressings (Gao et al. 2025). Furthermore, the physical characteristics of the formulation influence its ability to conform to irregular wound surfaces and facilitate appropriate interaction or penetration of therapeutic agents into the wound bed. Consequently, topical formulations should provide sufficient wound retention while allowing appropriate transport of bioactive molecules to the target tissue. In addition to these physical properties, advanced wound dressings may actively contribute to biological repair. For example, some hydrogel formulations promote angiogenesis, extracellular-matrix formation, collagen deposition, and cellular proliferation while simultaneously functioning as carriers for therapeutic agents. The PADM@MgC hydrogel reported by Bao et al. (Bao et al. 2024) is an example of a multifunctional hydrogel exhibiting angiogenic activity and supporting wound repair. Overall, the effectiveness of herbal topical therapies should therefore be evaluated not only according to the antimicrobial, anti-inflammatory, antioxidant, or regenerative activity of the incorporated plant-derived compounds, but also according to the adhesion, moisture regulation, exudate absorption, stability, controlled-release behavior, tissue interaction, and practical applicability of the delivery system in which they are administe28red (Garg et al. 2025), (Gobinath et al. 2026).

### Effects of herbal topical agents on wound-healing cells and cytocompatibility

The therapeutic suitability of herbal topical agents depends not only on their antimicrobial activity but also on their compatibility with the cells involved in normal wound repair. Wound healing requires coordinated interactions among keratinocytes, fibroblasts, endothelial cells, and immune cells, which collectively regulate re-epithelialization, extracellular-matrix deposition, angiogenesis, inflammation, and tissue remodeling (Peña and Martin 2024). Therefore, an effective herbal topical formulation should ideally reduce microbial burden while preserving or promoting the viability and function of these essential cell populations. Recent reviews indicate that several plant-derived phytochemicals can influence cell proliferation, migration, collagen synthesis, angiogenesis, oxidative stress, and inflammatory signaling during different phases of wound repair (Riaz et al. 2024), (Farasati Far et al. 2024). Keratinocytes are essential for re-epithelialization, and evidence from recent experimental studies indicates that selected phytoconstituents can promote keratinocyte migration and survival. For example, madecassoside, a major triterpenoid of *Centella asiatica*, enhanced wound closure in HaCaT keratinocytes and protected these cells against UVB-induced apoptosis and loss of viability, with involvement of AMPK/mTOR and ERK signaling (Sucharitakul et al. 2026). Similarly, Aloe vera has been reported to stimulate pathways associated with keratinocyte proliferation and migration and to increase growth factors involved in epithelial repair, including KGF-1, TGF-β, IGF-1, VEGF, and bFGF (Lee et al. 2025). Fibroblasts are responsible for extracellular-matrix deposition and collagen synthesis during the proliferative phase. Asiaticoside and madecassoside have been shown to enhance fibroblast proliferation and collagen synthesis and to modulate the TGF-β/Smad and MMP/TIMP pathways, supporting their potential role in tissue regeneration and remodeling (Wu et al. 2012). Curcumin-based formulations have likewise been associated with fibroblast proliferation, collagen deposition, granulation-tissue formation, angiogenesis, and re-epithelialization. Endothelial cells are particularly important for restoring vascularization and oxygen supply to injured tissue. Several natural compounds have demonstrated pro-angiogenic effects through modulation of VEGF and related signaling pathways, although the strength of evidence varies among individual phytoconstituents and experimental models (Riaz et al. 2024). Immune cells are also critical because a controlled inflammatory response is required for effective wound repair, whereas persistent macrophage and inflammatory-cell activation can delay healing. Phytochemicals such as curcumin, quercetin, kaempferol, and protocatechuic acid have been reported to modulate inflammatory signaling pathways, including NF-κB, MAPK, TLR4, and related cytokine networks, potentially facilitating resolution of excessive inflammation (Riaz et al. 2024). However, these biological effects are dependent on concentration, exposure time, formulation, extraction method, and cell type. Thus, antimicrobial activity alone should not be considered sufficient evidence of suitability for wound treatment. Herbal topical agents should additionally be evaluated for cytocompatibility using assays of cell viability, proliferation, migration, apoptosis, oxidative stress, and, where appropriate, endothelial angiogenic responses and immune-cell modulation. This integrated assessment is essential to determine whether a formulation can achieve effective microbial control at concentrations that preserve or promote the cellular processes required for physiological wound healing (Ansari et al. 2023). Despite the potential of topical herbal preparations as supportive approaches for wound management, their clinical translation is associated with several safety and quality-related limitations that should be considered. Allergic reactions and contact dermatitis are important concerns because wound application involves direct exposure of compromised skin to multiple phytoconstituents. Botanical ingredients, essential oils, resins, and other plant-derived constituents may act as sensitizers and can produce erythema, pruritus, inflammation, or allergic contact dermatitis (Gilissen et al. 2018). Furthermore, the use of herbal preparations on damaged or inflamed skin may increase the possibility of sensitization and local irritation. Another major limitation is batch-to-batch variability and inconsistency in phytochemical composition. The concentration and profile of bioactive constituents can vary considerably depending on plant species or chemotype, geographical origin, soil and climatic conditions, harvesting season, plant maturity, post-harvest processing, extraction solvent, extraction conditions, and storage. Such variability can subsequently influence the antimicrobial, anti-inflammatory, antioxidant, and wound-healing activities of the final preparation (Li et al. 2008). Overall, the development of topical herbal wound-healing products requires rigorous standardization, physicochemical and phytochemical characterization, microbial and contaminant testing, stability assessment, skin-irritation and sensitization studies, cytocompatibility evaluation, and investigation of compatibility with commonly used wound-care products before clinical application. These measures are essential to ensure that the therapeutic benefits of herbal preparations are accompanied by consistent quality, safety, and reproducible wound-healing efficacy (Govindaraghavan and Sucher 2015).

### Compare nanocomposite dressings with nanoparticles

Three-dimensional polymer networks incorporating nanoparticles into their structure are known as nanocomposite hydrogels. Biocompatible substances like polysaccharides (such as alginate, chitosan, and carrageenan), proteins (such as gelatin, keratin, and sericin), or synthetic polymers that fulfil the desired qualities for wound healing make up this polymer matrix. For example, maintain a moist environment, facilitate gas exchange, absorb wound exudates, provide mechanical support, and act as a physical barrier. The nanoparticles that are incorporated in these provide additional characteristics to them, like antimicrobial activities, bioactive agents, inflammation modulation, antioxidant properties, etc. The combination of the matrix and nanoparticle leads to synergistic effects that enhance the overall wound healing outcome. For example, CSLNs and *L. plantarum* were co-incorporated in the hydrogel dressing (Kaur Sandhu et al. 2023) and chitosan-based hydrogel loaded with silver nanoparticles and calendula extract (Rodríguez-Acosta et al. 2022).

### Synergistic, additive, and antagonistic interactions among herbal constituents

The biological effects of herbal formulations are influenced by interactions among their constituent phytochemicals, which may result in additive, synergistic, or antagonistic effects depending on the constituents, their relative concentrations, and their molecular targets. Recent experimental evidence provides quantitative support for synergistic interactions between herbal constituents. Gao et al. (Gao et al. 2025) demonstrated that the combination of honokiol, magnolol, and baicalin, three active constituents identified from Huangqin Houpo decoction, produced significantly greater anticancer activity than individual or two-component treatments in colorectal cancer models. Using ZIP synergy analysis, the authors reported strong synergistic interactions, with the combination showing a Combination Index (CI) of 0.215 in HCT116 cells and 0.236 in LoVo cells; according to their classification, CI values between 0.2 and 0.4 indicate strong synergy. The combination also produced greater inhibition of tumor growth in vivo and enhanced the response to anti-PD-1 immunotherapy, illustrating how complementary mechanisms of individual phytochemicals can contribute to an enhanced overall pharmacological effect. However, such synergy should not be generalized to all herbal combinations. In a 2025 systematic database study, Zhang et al. curated 1,055 interactions involving 331 phytochemicals and 244 anticancer drugs from 1,020 publications. Among these interactions, 760 interactions involving 307 phytochemicals enhanced anticancer-drug efficacy, whereas 41 interactions involving three phytochemicals reduced drug efficacy and seven interactions involving seven phytochemicals were associated with increased drug toxicity. These findings demonstrate that phytochemical combinations can produce beneficial synergistic effects but may also result in antagonism or enhanced toxicity, emphasizing the importance of experimentally establishing combination effects rather than assuming that multiple constituents will always act synergistically (Zhang et al. 2025a).

Although herbal products are often perceived as inherently safe because of their natural origin, this assumption is not necessarily justified, particularly when bioactive compounds are administered as concentrated extracts, essential oils, or nanoformulations. The safety profile of an herbal preparation depends on the plant species, chemical composition, dose, extraction method, route of administration, duration of exposure, and interactions with other medicines or constituents (Kongkaew et al. 2024). A 2024 systematic review and meta-analysis of pharmacovigilance data identified adverse-event reporting rates for herbal medicines ranging from 0.03% to 29.84%, with a median pooled estimate of 1.42%, and highlighted hepatotoxicity, renal injury, and allergic reactions among clinically relevant concerns (Cho et al. 2025). Similarly, an overview of 13 systematic reviews published in 2025 identified hepatobiliary adverse events as the most frequently reported organ-system effects, while nausea, diarrhea, and vomiting were among commonly reported symptoms associated with multi-herbal formulations. Particular caution is warranted with essential oils because their highly concentrated and lipophilic constituents can cause skin irritation, allergic reactions, phototoxicity, and, at sufficiently high exposures, neurotoxicity; therefore, concentration and route of administration are critical determinants of safety (Abdel-Hakeem et al. 2025). Furthermore, incorporation of herbal extracts or phytochemicals into nanoparticle-based delivery systems can alter absorption, tissue distribution, cellular uptake, and bioavailability, potentially changing both therapeutic efficacy and toxicity. Recent nanotoxicological literature indicates that nanoparticle-associated adverse effects may involve oxidative stress, inflammation, cellular damage, and organ-specific toxicity, with particle size, surface charge, composition, and exposure route influencing the biological response. Thus, the designation “natural” should not be considered synonymous with “non-toxic,” and concentrated extracts, essential oils, and nano-herbal formulations require appropriate dose optimization, physicochemical characterization, toxicological evaluation, and pharmacovigilance before their safety can be established (Philips and Theruvath 2024).

### Clinical applicability, stability, and scalability of herbal wound products

The clinical applicability of herbal wound products depends not only on their therapeutic efficacy but also on practical considerations such as shelf stability, storage, manufacturing reproducibility, cost, patient adherence, and scalability (Bansal et al. 2016). Plant-derived products may be susceptible to oxidation, hydrolysis, photodegradation, and changes in moisture or temperature, highlighting the importance of appropriate packaging, storage conditions, and stability testing in Table 5. Manufacturing reproducibility can be affected by variations in plant species, geographical origin, cultivation, harvesting, processing, and extraction methods; therefore, botanical authentication, standardized extraction procedures, chemical fingerprinting, marker-compound quantification, and quality-control testing are essential to minimize batch-to-batch variability. In addition, formulation characteristics such as adhesion, moisture retention, controlled release, mechanical stability, and ease of application can influence therapeutic performance, patient comfort, and adherence. Although medicinal plants may offer advantages in terms of local availability and potentially low-cost raw materials, the costs associated with extraction, purification, formulation, sterilization, quality control, packaging, and large-scale manufacturing should also be considered. Successful translation into clinical practice therefore requires scalable and reproducible manufacturing processes, appropriate stability and storage specifications, evidence of safety and quality, cost-effectiveness assessment, patient acceptability, and adequately designed clinical trials (Chen et al. 2025).

**Table 5:** Practical and Translational Considerations for the Clinical Application of Herbal Topical Wound-Healing Products

Practical Consideration	Key issues	Potential impact on clinical application	Recommended considerations/ solutions	References
Shelf stabilty	Plant-derived bioactive compounds may undergo oxidation, hydrolysis, photodegradation, or other changes during storage	Loss of antimicrobial, antioxidant, anti-inflammatory, or regenerative activity and reduced product shelf life	Perform accelerated and long-term stability studies; optimize packaging, temperature, moisture, oxygen and light protection	(Atanasov et al. 2021)
Storage requirements	Herbal preparations and formulations may be sensitive to temperature, humidity, oxygen, and light	Strict storage requirements may limit use, particularly in resource-limited settings	Establish appropriate storage conditions and develop formulations with improved thermal and chemical stability	(Chen et al. 2025)
Manufacturing reproducibility	Phytochemical composition can vary with species, geographical origin, cultivation, harvesting season, plant part, processing, and extraction method	Batch-to-batch variation may result in inconsistent efficacy and safety	Botanical authentication, standardized extraction, chemical fingerprinting, marker-compound quantification, and validated manufacturing procedures	(Atanasov et al. 2021)
Standardization and quality control	Complex extracts contain multiple active/inactive constituents and may contain contaminants or degradation products	Difficulty in establishing consistent dose, potency, purity, safety, and regulatory specifications	Establish chemical and microbiological quality-control parameters and specifications for identity, purity, potency, and contaminants	(Zhang et al. 2024)
Cost	Although medicinal plants may be inexpensive as raw materials, extraction, purification, formulation, sterilization, packaging, and quality testing can increase costs	Higher production costs may reduce accessibility and affordability	Optimize extraction and manufacturing processes and assess cost-effectiveness during product development	(Atanasov et al. 2021)
Patient adherence	Frequent application, unpleasant odor/appearance, irritation, discomfort, poor retention, or difficult dressing removal can reduce adherence.	Poor adherence may reduce real-world effectiveness despite demonstrated laboratory activity.	Develop comfortable, easy-to-apply, flexible and conformable formulations with an appropriate application frequency.	(Zhang et al. 2024)
Wound-surface adhesion	Insufficient adhesion may result in premature detachment, whereas excessive adhesion can damage newly formed tissue during dressing removal	Influences therapeutic contact time, tissue protection, comfort, and dressing-change requirements	Optimize adhesive properties according to wound type; consider controlled-adhesion or minimally adherent systems.	(Ahmed et al. 2025)
Controlled release	Rapid diffusion can cause loss of active compounds, whereas excessively slow release may limit therapeutic availability	Determines local concentration and duration of antimicrobial or regenerative effects	Optimize polymer composition, cross-linking, porosity, degradation, and stimulus-responsive properties.	(Zhang et al. 2024)
Formulation stability and mechanical integrity	Changes in swelling, degradation, mechanical strength, or structural integrity can affect dressing performance	Poor physical stability may reduce therapeutic efficacy and handling properties	Evaluate swelling, degradation, mechanical strength, self-healing, and storage stability	(Bansal et al. 2016)
Scalability	Laboratory formulations may use specialized materials or complex preparation procedures that are difficult to reproduce at industrial scale	Promising experimental products may fail during scale-up or become economically impractical	Develop simple, reproducible, cost-effective manufacturing processes and perform scale-up validation	(Ahmed et al. 2025)
Clinical validation	Many herbal wound products remain supported mainly by in vitro and animal studies	Laboratory efficacy may not translate into clinical effectiveness or safety	Conduct adequately designed clinical trials using standardized formulations and clinically relevant outcomes	(Chen et al. 2025)
Regulatory applicability	Complex herbal products can be difficult to characterize with respect to composition, dose, potency, quality, and safety	May complicate regulatory approval and clinical adoption	Establish clear specifications for botanical identity, purity, potency, stability, manufacturing quality, and safety	(Chen et al. 2025)

### Clinical Trials

Several clinical trials have investigated plant-derived products for wound infection and wound healing as requires support from reproducible in vitro and/or in vivo experimental studies, well-characterized phytochemical profiles, standardized preparations. Clinical trial NCT06690814 evaluated *Ficus septica*, *Pterocarpus indicus*, and *Curcuma longa* (turmeric) preparations for small infected cutaneous ulcers, comparing them with Savlon and no treatment; the study is particularly relevant because it directly examined plant-based interventions in infected wounds and assessed ulcer healing and wound-size reduction. NCT04453124 investigated *Ficus septica* sap as a topical treatment for cutaneous ulcers, comparing it with chlorhexidine and soap-and-water care, with clinical healing and wound microbiology assessed during follow-up. NCT01868412 compared Norway spruce (Picea abies) resin salve with medical honey in surgical wounds following vascular surgery, including eradication of cultured pathogenic bacteria as an important outcome. NCT03391310 evaluated Manuka honey for pressure ulcers in critically ill children, assessing healing time, treatment failure, and new wound infection; however, patients with established acute wound infection were excluded, making it more relevant to wound healing and infection prevention. NCT02819817 was designed to evaluate topical Aloe vera gel for traumatic wounds, including wound infection as an outcome, but the study was withdrawn without participant enrollment and therefore provides no clinical efficacy data. The detail information of clinical trial were summarise in Table 6.

**Table 6:** List Clinical Trials using Plant-based intervention in wound care

Clinical trial no.	Plant/ natural product	Condition	Status	Total participants	Main intervention	Major outcome
NCT06690814	*Ficus septica*, *Pterocarpus indicus*, *Curcuma longa*	Small infected cutaneous ulcers	Completed	370	Topical F. septica exudate, P. indicus exudate, or aqueous C. longa extract; compared with Savlon or no treatment	Ulcer healing and reduction in ulcer surface area at days 7 and 14
NCT04453124	*Ficus septica* sap	Small skin wounds / cutaneous ulcers, including prevention of bacterial ulcer development	Completed	150	*F. septica* sap vs. topical chlorhexidine vs. soap/water	Clinical healing and changes in wound microbiology over 21 days
NCT01868412	Norway spruce resin (Picea abies) and Manuka honey (Leptospermum scoparium)	Surgical wounds / wound infection after vascular surgery	Completed	40	Abilar 10% resin salve vs. medical honey (Activon)	Wound healing; eradication of cultured pathogenic bacteria; safety and cost
NCT03391310	Manuka honey (Leptospermum honey)	Pressure ulcers in critically ill children	Completed	100	Honey dressing vs. standard treatment	Time to healing; new-onset wound infection; treatment failure; ICU stay
NCT02819817	Aloe vera gel	Simple traumatic wounds	Withdrawan	0	Topical Aloe vera gel vs. ultrasound gel placebo vs. standard care	Time to epithelial healing; wound assessment; wound infection rate

## Conclusion

As per the available evidence, combining plant extracts or phytoconstituents with advanced wound-dressing materials, especially hydrogels and nanoparticle-based systems, exhibits promising applications for wound healing, including infected and diabetic wounds. The multifaceted biological activities of these compounds, together with the controlled-release and delivery capabilities of advanced dressing systems, may provide important therapeutic advantages. Moreover, most recent evidence is derived from several in vitro and clinical studies and substantial gaps that remains in between laboratory findings and clinical implementation. Therefore, standardized formulations, well-characterized active constituents, comprehensive safety and pharmacological evaluations, reproducible manufacturing, and well-designed clinical trials are required to establish their efficacy and safety before their widespread adoption in routine wound care.
